# Evaluating Knee Mechanisms for Assistive Devices

**DOI:** 10.3389/fnbot.2022.790070

**Published:** 2022-05-30

**Authors:** Shawanee' Patrick, Namita Anil Kumar, Pilwon Hur

**Affiliations:** ^1^Department of Mechanical Engineering, Texas A&M University, College Station, TX, United States; ^2^Department of Mechanical Engineering, Gwangju Institute of Science and Technology, Gwangju, South Korea

**Keywords:** knee mechanism, interaction forces, polycentric, migration, orthoses, rehabilitation, assistive devices, gait

## Abstract

State-of-the-art knee braces use a polycentric mechanism with a predefined locus of the instantaneous center of rotation (centrode) and most exoskeleton devices use a knee mechanism with a single axis of rotation. However, human knees do not share a common centrode nor do they have a single axis. This leads to misalignment between the assistive device's joint axis and the user's knee axis, resulting in device migration and interaction forces, which can lead to sores, pain, and abandonment of the device over time. There has been some research into self-aligning knee mechanisms; however, there is a lack of consensus on the benefit of these mechanisms. There is no research that looked purely at the impact of the knee mechanisms, either. In this article, we compare three different knee brace mechanisms: single axis (SA), polycentric with predefined centrode (PPC), and polycentric with a self-aligning center of rotation (PSC). We designed and conducted an experiment to evaluate different joint mechanisms on device migration and interaction forces. Brace material, weight, size, cuff design, fitment location, and tightness were consistent across trials, making the knee joint mechanism the sole variable. The brace mechanisms had no significant effect on walking kinematics or kinetics. However, the PPC brace had greater interaction forces on the top brace strap than the SA and PSC. The PSC and SA had significantly lower interaction forces on the bottom strap compared to the PPC brace. The PSC had significantly less migration than both the SA and PPC braces. These results show that a PPC mechanism may not be beneficial for a wide range of users. This also shows that the PSC mechanisms may improve mechanism alignment and lessen device migration.

## 1. Introduction

The human knee is not a simple pin joint; instead, the femur rotates and slides on the tibia as it flexes or extends (Morrison, 1970). This results in a joint with a varying center of rotation. At any time, the joint's axis is termed the Instantaneous Center of Rotation (ICR) and the locus of the ICR is called the centrode. Exoskeleton joint design typically requires that the joint's axis be coincident with the user's knee axis. Designing an exoskeleton knee joint that accurately tracks the user's ICR is a mighty task, and it is further compounded by the fact that the centrode is unique to the user. Although knee assistive devices have existed since the 1960s, the aforementioned challenge persists. In this article, we will investigate different solutions to this challenge in a human subject experiment.

### 1.1. Solutions to Knee Joint Design

The Single Axis (SA) joint knee mechanism is the simplest design to manufacture and actuate in powered devices. However, the misalignment between the device joint axis and the user's knee axis is unavoidable, which can lead to increased interaction forces and device migration (Regalbuto et al., 1989). High interaction forces may result in skin sores, additional pain, or injuries (Chen et al., 2016; Gorgey, 2018). Studies such as Pierrat et al. (2015); Anil Kumar et al. (2019); Serrancoli et al. (2019) have shown that interaction forces are strongly related to safety, comfort, and quality of walking with lower limb orthotics/exoskeletons.

**Figure 1:**
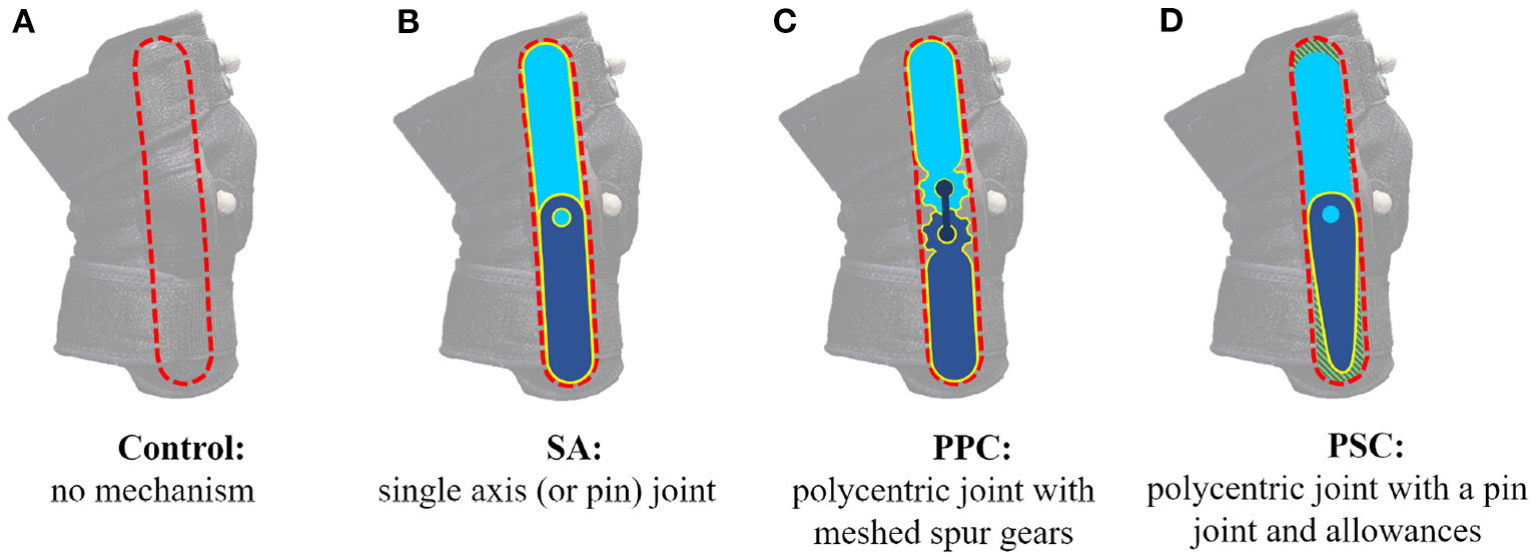
Knee brace mechanisms: **(A)** *Control* brace with no mechanism, **(B)** single axis (SA) brace with a single axis mechanism, **(C)** polycentric mechanism with a predefined centrode (PPC) brace with a polycentric mechanism having spur gears, and **(D)** polycentric mechanism with a self-aligning center of rotation (PSC) brace with a polycentric mechanism with allowances.

Some researchers have implemented polycentric knee mechanisms, which are of two types: (i) Polycentric mechanism with a Predefined Centrode (PPC) and (ii) Polycentric mechanism with a Self-aligning Center of rotation (PSC). PPC solutions either adopt a centrode that is believed to suit a diverse group of users (Bertomeu et al., 2007) or customize the centrode to the user (Supan, 2019). The most commonly implemented PPC mechanism has meshed spur gears with a third link connecting the centers of the gears (Lee et al., 2020) (also refer to Figure 1C). Other PPC joint designs employ cam mechanisms (Bertomeu et al., 2007; Kapci and Unal, 2019). Despite efforts to establish a generalized centrode for a large user base, discrepancies are to be expected. On acknowledging this, some researchers chose to customize the gear or cam mechanism (thereby the associated centrode) to the user (Supan, 2019). While the performance with customized joints is expected to be better, the process of designing and manufacturing custom joints can be highly demanding. Researchers, thus, support using PSC joints which are believed to suit a diverse group of users. Though instances of PSC joints exist in the literature (Stienen et al., 2009; Celebi et al., 2013; Cai et al., 2016; Choi et al., 2016), their public usage is very limited with the likely reason being a lack of consensus and data on the performance of PSC joints. In this article, we will strive to resolve this dilemma by comparing all three types of joint designs (i.e., SA, PPC, and PSC).

### 1.2. Evaluating Knee Brace Mechanisms

Studies such as Brownstein (1998) have examined how different knee brace designs impact migration. While the designs belonged to the PPC category, they all varied in size, material, nature of fit, and cuff design. Work by Regalbuto et al. (1989) evaluated different joint mechanisms by observing the interaction forces at the straps of custom brace cuffs. However, the study did not look at the self-aligning joints or device migration. To our knowledge, no study has compared different joint mechanisms on the basis of interaction forces and migration. Moreover, the studies (Regalbuto et al., 1989; Brownstein, 1998) do not account for variances in the brace fitment (i.e., tightness of the cuffs) at the beginning of each trial, which heavily influences the performance of the brace. In order to perform a controlled analysis of the joint mechanisms, we must hold constant the material, weight, size, cuff design, and tightness of fit. Current experimental protocols do not account for the impact of the previously mentioned variables and limit their performance metrics to primarily device migration. Thus, there is significant room for improvement in designing experiment protocols for joint mechanism comparison. In this article, we will fill this gap in knowledge by proposing a systematic experiment protocol that evaluates both device migration and interaction forces.

Our primary contributions include: (i) the experiment protocol for evaluating different knee mechanisms, (ii) a novel PSC design inspired by Cai et al. (2016), and (iii) evidence that will help identify the superior joint mechanism design. The article is organized as follows. Section 2 presents the experiment setup, protocol, and details on the recruited subjects. The results are presented in Section 3 followed by the discussion in Section 4. The final section consists of our concluding remarks.

## 2. Materials and Methods

We designed an experiment to evaluate different joint mechanisms on device migration and interaction forces. The variables accounted for were brace material, weight, size, cuff design, fitment location, and tightness. The first four and the last two variables were considered in the experiment setup and testing protocol, respectively.

### 2.1. Participants

Twelve healthy subjects were recruited. The method of determining outliers has been detailed in Section 2.3. Out of the 12 subjects, one was deemed an outlier and another subject was omitted from the study due to a failure in data collection. The results presented pertain to 10 healthy participants (age 28 ± 2.5 years, mass 70.5 ± 11.2 kg, height 171.3 ± 5 cm, 7 men and 3 women). Individual participant details can be found in Table 1. The experimental protocol was explained beforehand, and each subject signed an informed consent approved by Institutional Review Board (IRB) at Texas A&M University (TAMU IRB2018-0837D).

**Table 1:** Individual details for the final 10 participants.

**Participant**	**Mass (kg)**	**Height (cm)**	**Age**	**BMI**	**Knee width (cm)**	**Sex**
1	59.3	170.2	28.0	20.5	10.1	M
2	51.0	164.0	28.0	19.0	9.3	F
3	74.7	180.3	27.0	23.0	10.4	M
*4	85.3	177.8	26.0	27.0	10.2	M
5	65.2	172.7	28.0	21.9	9.7	M
6	69.7	169.5	27.0	24.2	11.2	F
7	71.0	170.0	32.0	24.6	11.2	M
8	79.9	172.7	30.0	26.8	11.3	F
9	63.2	166.0	23.0	22.9	9.8	M
10	85.3	170.0	30.0	29.5	12.0	M
Average	70.5	171.3	27.9	23.9	10.5	F - 3
Standard deviation	11.2	4.9	2.5	3.2	0.9	M - 7

*Not included in kinetic and kinematic analysis.

### 2.2. Experimental Setup

**Figure 2:**
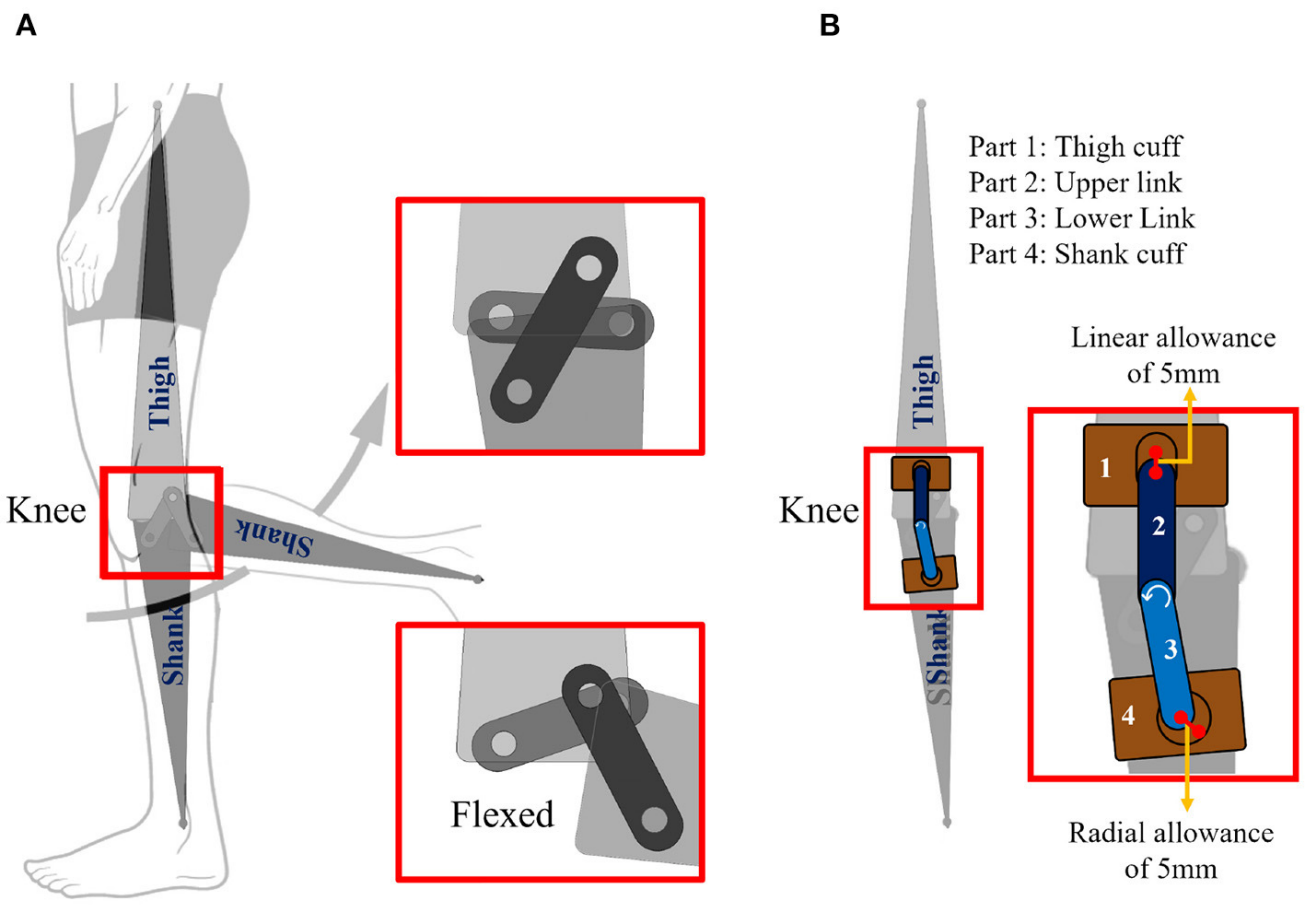
**(A)** Four-bar simulation of the human knee. **(B)** Proposed PSC mechanism.

Compression braces, such as VIVE (Vive, 2021), consist of a fabric sleeve with slots on both sides of the knee for a geared PPC mechanism. Figure 1A shows the VIVE brace and highlights the slot for the mechanism (*mechanism-slot*). Such braces have the benefit of the mechanism being removable, allowing us to swap and test different mechanisms. We procured four VIVE braces and designed different Polylactic acid (PLA) 3D printed mechanisms to fit the brace's *mechanism-slot*. Figure 1 presents all four braces. The brace in Figure 1A had no constraining mechanism and served as our control case, while Figure 1B was the SA version. Figure 1C was the PPC mechanism that was included with the VIVE brace. Figure 1D is a novel PSC joint consisting of a single axis joint at the knee, a linear allowance at the top of the *mechanism-slot* (hashed green region), and a radial allowance at the bottom of the *mechanism-slot* (hashed green region). This design draws inspiration from the easy-to-manufacture design proposed in Cai et al. (2016). The design was optimized through kinematic analysis of different brace PSC designs using Solidworks. Said analysis involved a four-bar approximation of the human knee in the sagittal plane (refer to Figure 2). The length of the linkages was the average of the results reported in Pons et al. (2007). Figure 2B shows the final PSC design acting in parallel with the human knee simulation. The thigh cuff was constrained to not move while the shank cuff was allowed to slide (or migrate) along the limb. The PSC design was optimized as follows: (i) vary the magnitude of allowance (ii) flex the simulated human knee to 70° and measure the device migration along the shank (iii) repeat steps (i) and (ii) until the device migration is minimal. The optimal design shown in Figure 1D has allowances of 5 mm. Notice that the SA, PPC, and PSC braces only vary in the joint mechanism.

**Figure 3:**
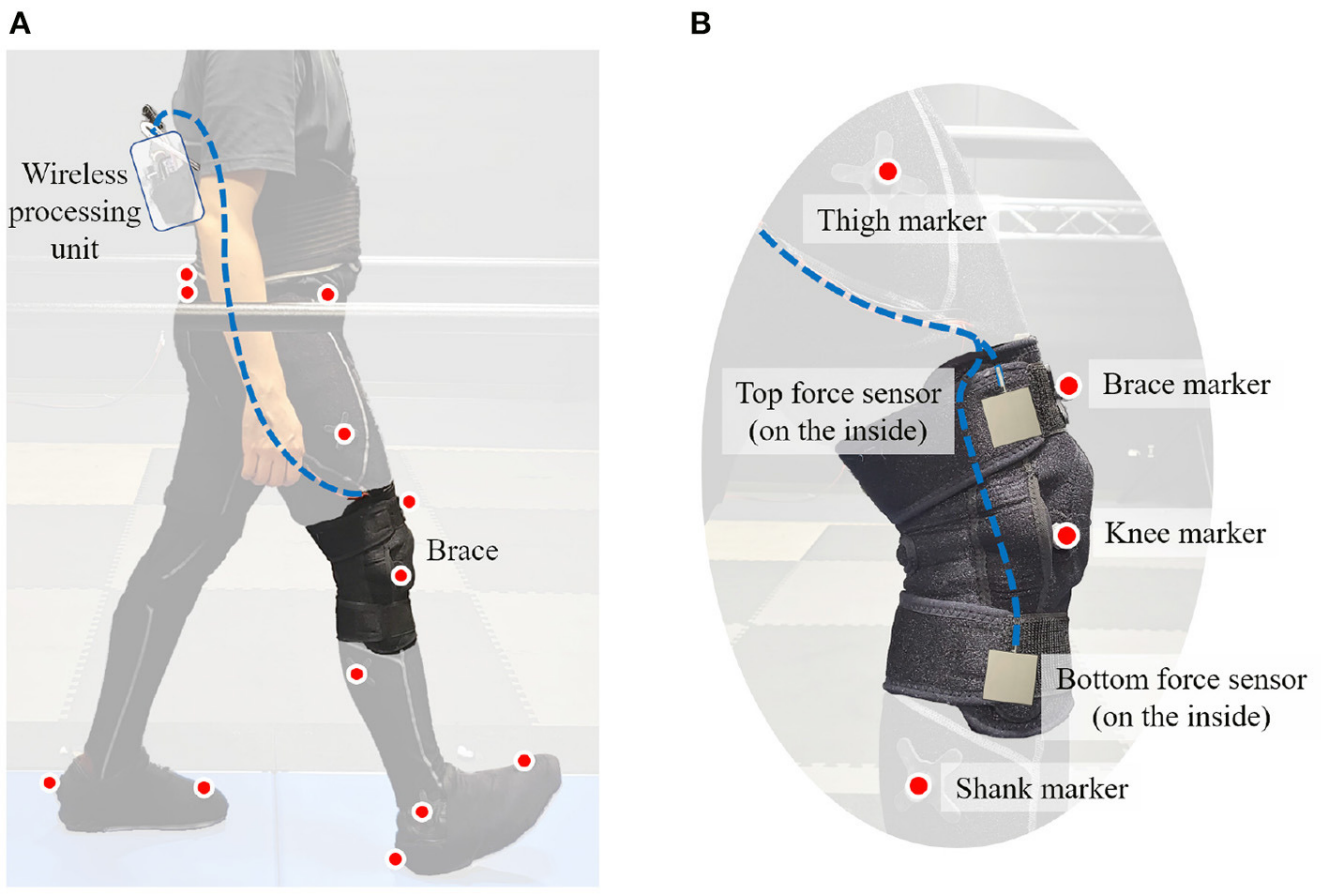
Experiment setup: **(A)** Subject with markers and a brace, **(B)** markers and sensors mounted on the brace.

All braces were fitted with two Tekscan FlexiForce A502 flexible force sensors (Figure 3B) which served to measure the interaction forces at the user's thigh and shank. These locations were chosen for two reasons: (i) they are along with the knee brace straps–where interaction forces are expected to be the highest; (ii) the mounted force sensors would always be in contact with the participant's limb. Unlike the sides of the brace, the front section is not always in contact with the participant's limb, making this spot not ideal for measuring interaction force. Specifically, this section of the brace separates from the limb (forming a gap) during knee flexion. The sensor readings were collected and transmitted using a wireless processing unit consisting of an Arduino Micro and XBee Pro wireless module. The unit was affixed to a vest worn around the participant's torso. The receiver unit consisted of an XBee Pro wireless module and an Arduino Uno, which transmitted the received data to a computer for storing. The experiment included walking on an instrumented treadmill (Force-sensing treadmill, AMTI, Watertown, MA AMTI, 2021) in a motion capture facility that uses 46 motion capture cameras (Vantage, Vicon Motion Systems, Oxford, UK Vicon, 2021). Reflective markers were placed on bony landmarks of the pelvis, lateral knee joint, toe, heel, and ankle. Additional markers were placed on the thigh, tibia, and front of the brace. The marker placement can be seen in Figure 3A.

### 2.3. Experiment Protocol

**Figure 4:**
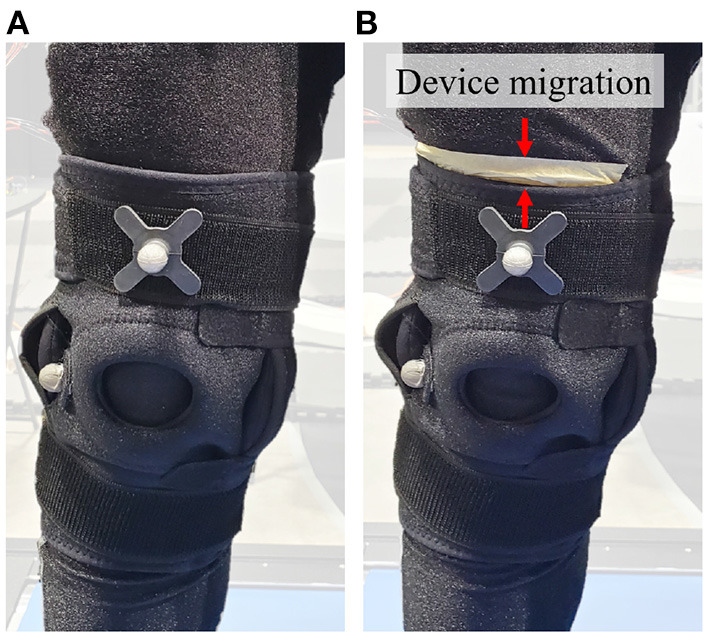
Device Migration, **(A)** the brace at the beginning of the trial, **(B)** the brace at the end of the trial with the white tape marking the reference for measuring device migration. Red arrows show device migration as labeled on the figure.

Each participant was instructed to wear workout leggings or tights and tennis shoes. The participants were then asked to wear the Control brace tightened to their comfort. Once worn, the brace position was marked with tape on the thigh. Each participant was then given a period of 2 min to get accustomed to the brace, during which they were asked to walk at a comfortable pace and raise their knee. After the 2-min period, device migration was measured by the distance from the top of the brace to the top of the tape (refer to Figure 4). If the device migration exceeded 1 cm, the brace was re-attached and the process was restarted. If participants failed the <1 cm device migration requirement after three attempts, they were ruled as an outlier and were omitted from the study. Such participants were expected to experience even larger device migration and consequently discomfort during the rest of the trial which consisted of higher paced walking trials and several knee raises. Typically, participants with a more tapered lower limb (i.e., a larger ratio of the above knee to below knee diameter) were found to be outliers. Once a suitable fitment was determined, the position of the brace was marked using the tape. All other braces that followed were mounted at the same position, fixing the point of fitment across all trials.

The order of the constrained braces (i.e., SA, PPC, and PSC) that followed was randomized. During the first constrained brace trial, the tightness of fit was measured using the force sensors. The force readings at the bottom and top force sensors were referred to as $f_{bottom}^{0}$ and $f_{top}^{0}$. The constrained braces that followed were then fitted to within ±1 N of said measured forces. While measuring forces, participants were asked to stand erect and still. This procedure standardized the tightness of fit across all constrained braces. Note that the forces were not measured for the Control brace because the absence of a constraining mechanism always resulted in a lower force reading.

**Figure 5:**
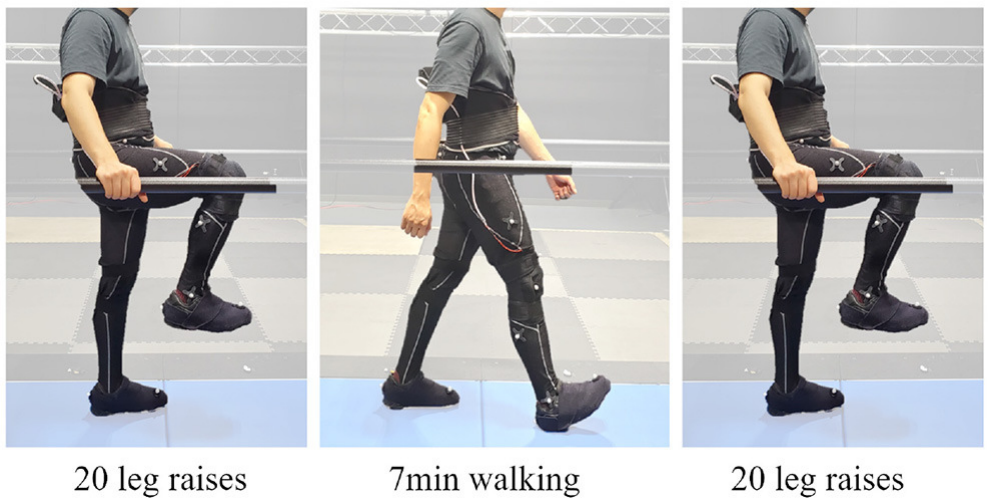
Each trial consisted of 20 leg raises, followed by 7 min of walking at 1.23 m/s speed, and concluded with another 20 leg raises.

Once fitted with each brace, the participants were asked to perform an exercise regime that included 20 knee raises, 7 min of fast walking at 1.23 m/s, and 20 more knee raises (refer to Figure 5). Motion marker data were collected before knee raises, during walking (to monitor walking quality), and after knee raises. The force sensor readings were gathered throughout the trial. Due to the data being used to assess the potential impact of walking assistive devices, the exercise routine was designed not to be labor intensive. The goal was to see the impact primarily during walking. Device migration was measured after the exercise routine for each brace device.

### 2.4. Metrics and Data Analysis

Three metrics were used to compare the knee braces: (i) device migration; (ii) maximum interaction force at the bottom and top force sensors; (iii) knee angles and moments during walking.

The device migration, *M*_*i*_, for each constrained brace (*i* = SA, PPC, PSC) was defined as follows


(1) $$\begin{array}{l} M_{i}=\frac{m_{i}-m_{Control}}{m_{Control}} \end{array}$$

where *m*_*i*_ is the raw (un-normalized) migration for each constrained brace (*i* = SA, PPC, PSC) and *m*_*Control*_ is the migration with the Control brace. The normalization process helps account, to some extent, for the impact of the compression sleeve on device migration, leaving behind the impact of the mechanism alone. The set of normalized migration values for each brace, across all subjects, was checked for normality using the Shapiro-Wilk test (α = 0.05, *scipy*'s *stats* library for Python). One-way repeated measures ANOVA was used to find the effect of the knee mechanism on device migration (α = 0.05, the *statsmodels* library for Python). *Post-hoc* tests used Fisher's least significant difference. Note that the device migration with the constrained mechanisms was not compared against the Control brace. Device migration with the Control brace is known to be lesser than the constrained ones and the objective of this article is to compare different constraining mechanisms.

The force values were first filtered using a Butterworth low pass filter with a cut-off frequency of 10 Hz, following which the maximum value was determined. Let $f_{bottom}^{*}$ and $f_{top}^{*}$ be the maximum force values at the bottom and top sensor, respectively. These values were then normalized for each constrained brace as follows.


(2) $$\begin{array}{l} F_{bottom}^{*}=\frac{f_{bottom}^{*}-f_{bottom}^{0}}{f_{bottom}^{0}} \end{array}$$

$F_{top}^{*}$ was calculated in a similar manner. Similar to *M*_*i*_, the set of all normalized force values was also checked for normality. A significant effect of the mechanism on force values was found *via* one-way repeated measures ANOVA, followed by the *post-hoc* tests with Fisher's least significant difference.

The joint angles and moments were derived using motion capture and forces collected with the AMTI instrumented treadmill and processed with the Vicon Nexus analysis system. The moments and angles were averaged for each participant for 30 s of the 7-min walking trial in each brace. The braced knee angle is determined to be the angle between the thigh and shank segments with the leg fully extended being 0 degrees. The range of motion for each braced knee was termed the difference between the maximum and minimum knee angle in each walking trial. These values were, averaged across all participants for each brace. The result was called the average range of motion. The braced knee moments were derived using inverse dynamics with the Vicon Nexus Plug-in Gait Model, after which the peak sagittal plane knee moments were determined. We checked if the nature of the brace mechanism impacted the peak knee moments and the knee ranges of motion using one-way repeated-measures ANOVA.

## 3. Results

**Figure 6:**
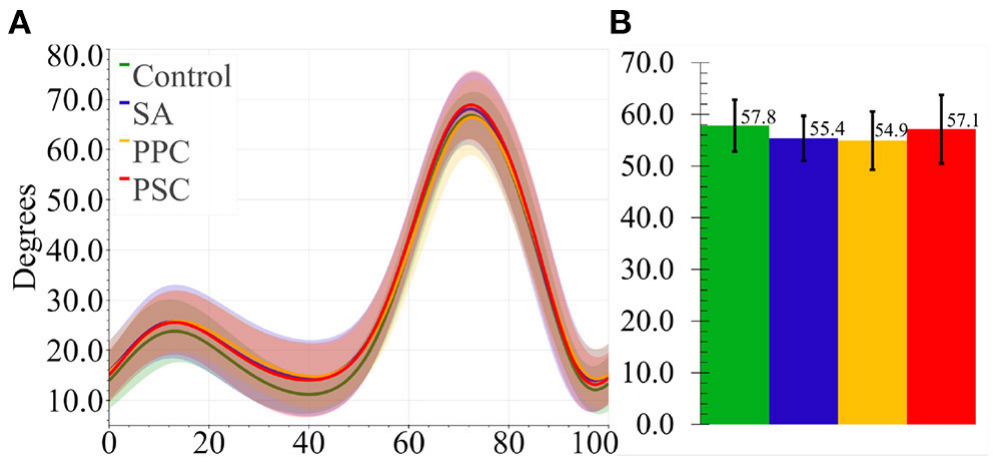
**(A)** Average knee angles for all four braces. The shaded region represented 1 SD. **(B)** Average knee range of motion for all braces. The ticks represent 1 SD.

**Figure 7:**
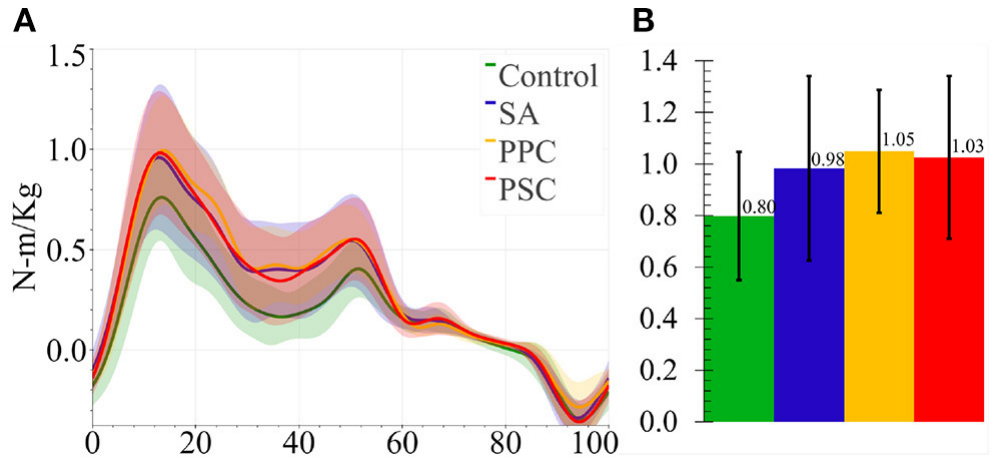
**(A)** Average knee moments for all four braces. The shaded region represented 1 SD. **(B)** Average peak knee moment for all braces. The ticks represent 1 SD.

**Figure 8:**
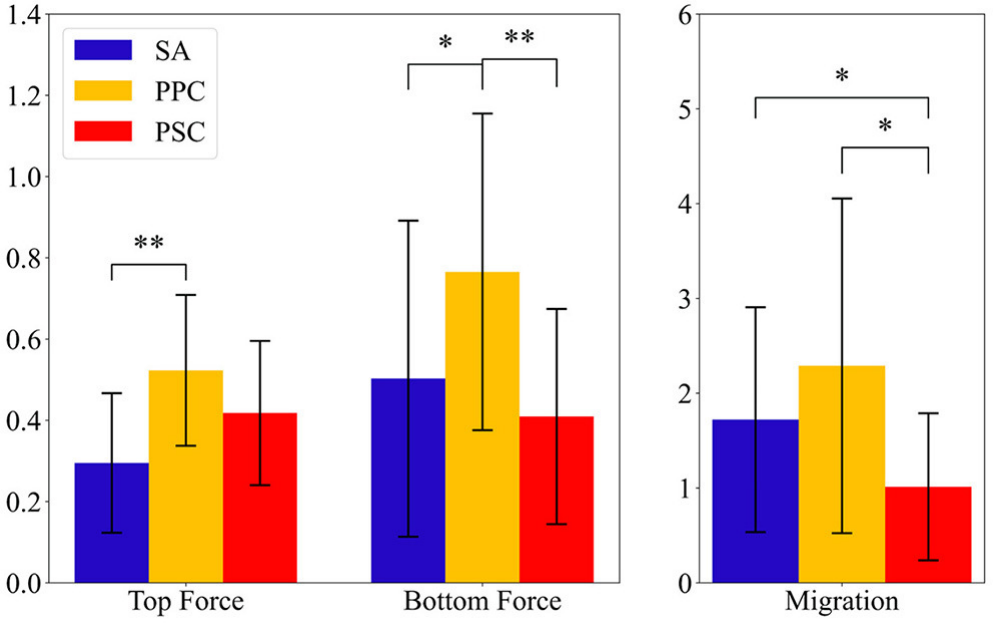
Average interaction force at the top and bottom force sensors and average device migration results. The ticks represent 1 SD. The symbol * signifies *p* < 0.05 and ** implies *p* < 0.005.

The following subsections present the results for the final 10 subjects in Table 1. Figures 6, 7 present the knee angle and knee moment results, respectively. The normalized device migration and interaction force values have been shown in Figure 8.

### 3.1. Kinematics and Kinetics

Data from Participant 4 was not processed for biomechanical analysis due to an error in the data collection, leaving 9 participants' knee angles and moments to be analyzed. The ANOVA test revealed that the bracing mechanism did not significantly impact the knee range of motion (*p* = 0.51) (refer to Figure 6) nor the knee moments (*p* = 0.276) (refer to Figure 7).

### 3.2. Brace Migration and Interaction Forces

The Shapiro–Wilk test revealed the normality hypothesis cannot be dismissed for migration data (*p* > 0.109 across all braces), top force sensor readings (*p* > 0.205 across all braces), and bottom force sensor readings (*p* > 0.135 across all braces). The one-way repeated measures ANOVA revealed that the type of mechanism significantly affects device migration (*p* = 0.0043), top force sensor readings (*p* = 0.007), and bottom force sensor readings (*p* = 0.0029). The SA and PPC brace had significantly more migration than the PSC brace with *p* = 0.022 and *p* = 0.007, respectively. Although the device migration with SA was lower than that of PPC, the difference was not significant. The interaction forces on the top of the PPC brace were found to be significantly greater than the SA brace (*p* = 0.004). The interaction forces on the bottom strap for the PPC brace were found to be significantly greater than both the SA (*p* = 0.016) and PSC braces (*p* = 0.005). These results can be seen in Figure 8.

## 4. Discussion

The brace type had no significant effect on the knee range of motion. This showed that none of the braces significantly altered walking gait kinematics. On the other hand, the knee moments with the Control brace were significantly lower than those with the other braces, which can be attributed to the absence of a constraining mechanism in the Control brace. In other words, the participants had to exert additional knee moments or work to overcome the constraints. Among the constrained mechanisms, no significant differences were observed. Thus, any observations made regarding device migration and interaction forces are solely due to the nature of the constraining mechanism and not the walking kinematics or kinetics.

In regards to device migration, SA performed better–but not significantly better–than PPC. The SA brace did however result in significantly lower interaction forces than PPC at both the top and bottom force sensor. We may infer that having a polycentric design alone is insufficient to perform better than SA mechanisms. However, the same polycentric design could perform better with certain types of knees over others. Going forward, we wish to investigate the relationship between knee widths and the performance (in terms of migration and interaction forces) of PPC mechanisms. If a relationship does exist, designers can use it to customize PPC designs to sections of the user population.

The PSC mechanism had significantly lower device migration than both SA and PPC, proving the benefits of self-aligning mechanisms. It also registered lower interaction forces than PPC at both the top and bottom force sensors, with the one at the bottom being significantly different. On the other hand, the interaction forces with PSC were not significantly different from those of SA. We believe that the force readings pertaining to PSC were an overestimate. The design of the PSC mechanism is such that it moves within the *mechanism-slot*, which can incur additional shearing forces. It is, thus, likely that the actual interaction forces with PSC are lower than the ones observed in this study. Our working hypothesis is that the interaction forces and device migration are correlated and that the PSC would outperform both SA and PPC per both metrics. Future study includes designing braces wherein the mechanism is placed away from the force sensors.

We also wish to improve the force sensing mechanism. The current mechanism only measures forces at the side of the thigh and shank. Studies such as Rossi et al. (2010) have measured forces around the limb using multiple pressure sensors along the curvature of the strap. Finally, we also hope to expand the study to include participants with more tapered limbs (i.e., greater ratio of above knee diameter to below knee diameter).

## 5. Conclusion

We propose an experiment protocol and analysis that compares the impact of knee mechanisms on interaction forces, migration, knee angles, and moments. This experiment protocol standardized the weight, material, and tightness of straps across all mechanisms. We compared three mechanisms: (i) Single axis (SA), (ii) Polycentric joint with a Predefined Centrode, and (iii) Polycentric joint with a Self-aligning Center of rotation (PSC). Although initially thought to increase interaction forces and migration, the SA mechanism produced consistently fewer interaction forces than the PPC mechanism. Thus, PPC mechanisms are not guranteed to lessen the mismatch between the mechanism and the user's knee. The PSC mechanism resulted in the least migration out of all the mechanisms. The forces with PSC were, significantly less than the PPC brace on the bottom strap. The significantly lesser migration of the PSC brace shows that it can assist in reducing the joint mismatch between the mechanism and knee. This gives us evidence supporting the use of PSC mechanisms in orthotics and exoskeletons. If researchers continue to use PPC mechanisms there needs to be further research on customizing the joint to improve alignment and overall performance.

## Data Availability Statement

The raw data supporting the conclusions of this article will be made available by the authors, without undue reservation.

## Ethics Statement

The studies involving human participants were reviewed and approved by Texas A&M Institutional Review Board. The patients/participants provided their written informed consent to participate in this study.

## Author Contributions

SP and NA contributed equally to this work. PH was the PI for this study. All authors contributed to the article and approved the submitted version.

## Conflict of Interest

The authors declare that the research was conducted in the absence of any commercial or financial relationships that could be construed as a potential conflict of interest.

## Publisher's Note

All claims expressed in this article are solely those of the authors and do not necessarily represent those of their affiliated organizations, or those of the publisher, the editors and the reviewers. Any product that may be evaluated in this article, or claim that may be made by its manufacturer, is not guaranteed or endorsed by the publisher.
